# Expression of CD98hc in Pancreatic Cancer and Its Role in Cancer Cell Behavior

**DOI:** 10.7150/jca.70500

**Published:** 2022-04-18

**Authors:** Daniela Bianconi, Elisabeth Fabian, Merima Herac, Markus Kieler, Johannes Thaler, Gerald Prager, Matthias Unseld

**Affiliations:** 1 Division of Oncology, Comprehensive Cancer Center, Department of Medicine I, Medical University of Vienna, Vienna, Austria; 2 Division of Gastroenterology and Hepatology, Department of Medicine III, Medical University of Vienna, Vienna, Austria; 3 Department of Pathology, Medical University of Vienna, Austria; 4 Institute for Vascular Biology, Center for Physiology and Pharmacology, Medical University Vienna, Vienna, Austria; 5 Division of Hematology, Department of Medicine I, Medical University of Vienna, Vienna, Austria; 6 Division of Palliative Medicine, Department of Medicine I, Medical University of Vienna, Vienna, Austria

**Keywords:** CD98hc, pancreatic ductal adenocarcinoma, PANC-1, BxPC-3

## Abstract

**Background:** Cluster of differentiation 98 heavy chain (CD98hc) is a transmembrane protein, which functions both as a coreceptor of ß-integrins, enhancing intracellular integrin-dependent downstream signaling, and as a transporter of branched-chain and aromatic amino acids. As such, it is pivotal in cell cycle regulation and protection of oxidative, nutritional and DNA replication stress. Overexpression of CD98hc occurs widely in cancer cells and is associated with poor clinical prognosis. The role of CD98hc in pancreatic cancer remains to be elucidated. The aim of this study was to determine the expression of CD98hc in pancreatic ductal adenocarcinoma and to define its potential functional role in cancer cell biology.

**Methods:** Immunohistochemical staining for CD98hc was performed on 222 tissue samples of patients with pancreatic ductal adenocarcinoma. The pancreatic cancer cell lines PANC-1 and BxPC-3 were used to determine the effect of CD98hc expression on cancer cell behavior using cell adhesion, cell trans-migration and cell spreading assays. Flow cytometry was performed to study the rate of apoptosis after detachment or serum starvation. shRNA-lentiviral constructs were used to knock down or reconstitute full length or mutated CD98hc.

**Results:** Up to 20% of pancreatic ductal adenocarcinomas express CD98hc in the acinar cells (13%) and islet cells (20%) embedded in tumor tissue. Although expression of CD98hc in tumor tissue was not associated with a particular tumor stage or grade, our data show a trend towards longer overall survival of pancreatic cancer patients without CD98hc expression as compared to those with immunohistochemical positivity. *In vitro* downregulation of CD98hc in the pancreatic cancer cell lines PANC-1 and BxPC-3 significantly inhibits cell proliferation (p<0.05), self-renewal (p<0.05) and anchorage-independent growth (p<0.05).

**Conclusion:** CD98hc is expressed in a remarkable percentage of pancreatic ductal adenocarcinomas. Due to its important role in cell behavior and malignant cell transformation, it may be a promising molecular target for potential new therapeutic approaches in pancreatic cancer in the future.

## Introduction

Cluster of differentiation 98 heavy chain (CD98hc, also known as SLC3A2, 4F2, FRP1) is an ubiquitously expressed part of heteromeric amino acid transporters (HATs) 1 and is involved in cell proliferation under physiological and pathological conditions 2,3,4. HATs are disulfide-bound heterodimers composed of a heavy subunit from the SLCs family (CD98hc) or rBAT (SCL3A1) and a light L-type amino acid transporter (LAT) from the SLC7 family 1. CD98hc-associated transporters are transmembrane proteins with two well-established roles: While CD98hc behaves as a coreceptor of ß-integrins and amplifies integrin-dependent downstream signaling via direct binding with cytoplasmic tails of 3 integrin subunits 5,6,7, the light chain (LAT1, LAT2, xCT, y^+^LAT1, y^+^LAT2 or asc1) mediates the transport of branched-chain and aromatic amino acids, conferring substrate specificity to the heterodimer 8. Under physiological conditions, CD98hc expression is limited to proliferating cells 9 and is required for clonal expansion of T cells and B cells 10,11. However, a growing body of evidence shows that CD98hc is overexpressed in various cancer cells and promotes malignant cell transformation and progression 6,12,13 by driving integrin-dependent cancer cell behavior 14 and metastatic potential 15,16. In addition, the amino acid transport function of CD98hc is suggested to play a crucial role in the growth, proliferation and survival of cancer cells, as well as in the formation of metastases 17,18. Rapidly proliferating cells extensively reprogram metabolic pathways to meet their increased demands for amino acids, which are not only used for protein synthesis but also serve as nitrogen and carbon sources for the synthesis of nucleotides, amino sugars and glutathione 19,20,21. Cancer cells are able to upregulate their nutritional and antioxidative capacity by overexpression of CD98hc-LAT1 and CD98hc-xCT (cystine/glutamate transporter) 3,22. Knockout of CD98hc in fibroblasts leads to cell death by ferroptosis 23,24, attributed to the loss of CD98hc-xCT, a transporter that sustains cellular redox homeostasis by uptake of cyst(e)ine, which is necessary for the biosynthesis of glutathione 25,26. Nutritional status regulates cell cycle progression in part by controlling protein synthesis via the mammalian target of rapamycin complex 1 (mTORC1) 27,28. In addition, also nucleotide biosynthesis has strict energetic and nutritional requirements. Thus, shortage of branched-chain and aromatic amino acids caused by CD98hc ablation phenocopies the inhibition of mTORC1 signaling. Moreover, it triggers a dramatic reduction in the nucleotide pool, which leads to replicative stress in cells as reflected by the increased DNA damage response, S-phase delay and diminished rate of mitosis 21. This suggests that CD98hc and an adequate availability of the branched-chain and aromatic amino acids are pivotal in cell cycle regulation and protection against oxidative, nutritional and DNA replication stress 21.

In renal cancer cells, a targeted loss of CD98hc blocks tumorigenic potential 14, and in cisplatin-resistant ovarian cancer cells CD98hc increases anti-tumor drug sensitivity 29. Together with ß1-integrin and transient receptor potential vanilloid 4 (TRPV4), CD98hc further forms the molecular basis for transmembrane mechanotransduction and the rapid induction of cellular calcium influx 30. Due to this crucial role of CD98hc in cell proliferation, signaling and metabolism as well as formation of metastases, it may serve as a specific molecular target for the development of new therapeutic strategies in different cancers.

The role of CD98hc in pancreatic cancer, which is a malignancy characterized by poor prognosis and limited therapeutic options 31,32, has not been established. The aim of this study was to determine the expression of CD98hc in pancreatic ductal adenocarcinoma and to define its potential functional role in cancer cell behavior to furnish data for potential new therapeutic approaches in this disease.

## Materials and Methods

This study examined 222 paraffin-embedded tissue samples of patients with pancreatic ductal adenocarcinoma. Tumors classified according to the TNM system from well-characterized patients (Table 1) were derived from the tissue bank of US Biomax (tissue microarrays HPan-Ade180Sur-01 and HPan-Ade150CS-01-BX). Male and female patients over 18 years of age, with pancreatic ductal adenocarcinoma (with or without lymph node metastases or secondary blastomatous lesions) who received surgery for the managment of their pancreatic cancer but without neoadjuvant therapy were enrolled in the study. Patients younger than 18 years of age without pancreatic ductal adenocarcinoma (e.g. pancreatic neuroendocrine tumors) or with pancreatic ductal adenocarcinoma who had already undergone neoadjuvant therapy or surgergy for their pancreatic cancer earlier, or patients presenting with a second malignacy were exluded from the study. In addition, normal adjacent pancreatic tissue of 141 samples and five fetal paraffin-embedded pancreatic tissue samples (aged from 19 to 24 weeks) were investigated in this study (tissue microarrays FeOrg-N090-01, BN961a). Since this study was done on tissue samples derived from a tissue bank, approval of the local ethics committee was not required.

### Immunohistochemistry

For immunohistochemical staining, sections were dewaxed and rehydrated as follows: 10 min in incubation in xylene I (Riedel-de Haën™, Honeywell, Seelze, Germany), 10 min incubation in xylene II (Riedel-de Haën™, Honeywell, Seelze, Germany), 5 min in 100% ethanol, 5 min in 70% ethanol and 5 min in water. Endogenous peroxidase activity was blocked by using the Dual Endogenous Enzyme Block (Dako EnVision™ + Dual Link System-HRP; Dako, Carpinteria, California, USA). Epitope retrieval was accomplished in 0.01 M citrate buffer (pH: 6.0, 10x; Sigma-Aldrich, St. Louis, Missouri, USA). Sections were stained for CD98hc using the primary antibody CD98 (clone C-20; 1:200; Santa Cruz Biotechnology, INC). The secondary antibody used was the polyclonal rabbit anti-goat immunoglobulin/HRP (Dako, Carpinteria, California, USA) in a 1:100 dilution in phosphate buffer saline (PBS). Counterstaining was performed by using a 1:5 dilution of Mayer's hemalum solution (Merck, Darmstadt, Germany) and mounted with Aquatex® (Merck, Darmstadt, Germany). Positive and negative controls were included in each staining batch. A section, in which all the tumor cells were positive for CD98hc served as a positive control. For the negative controls, the primary antibody was omitted. To assess CD98hc cell membrane staining, slides were examined under a microscope and scored by a pathologist. Scoring: (0) no staining, (+1) incomplete membrane staining or complete membrane staining in less than 10% of cells, (+2) complete membrane staining that is non-uniform in at least 10% of cells, or intense complete membrane staining in up to 30% of tumor cells, and (+3) uniform intense membrane staining of more than 30% of invasive tumor cells. Immunohistochemistry was defined as positive from score 1-3 and termed as negative at score 0.

### Cell culture

Pancreatic cancer cell lines PANC-1 and BxPC-3, obtained from the American Type Culture Collection (ATCC), were cultured in RPMI 1640 medium (Gibco, Life Technologies™, Carlsbad, California, USA) supplemented with 10% fetal bovine serum (FBS) and 5% penicillin/streptomycin (Gibco, Life Technologies™, Carlsbad, California, USA), and incubated at 37°C with 5% CO_2_. When cells reached 80% confluence, they were passaged using 0.5% trypsin-ethylenediaminetetraacetic acid (EDTA) (Gibco, Life Technologies™, Carlsbad, California, USA). HEK-293T cells, obtained from the ATCC, were cultured in DMEM (Dulbecco's Modified Eagle's Medium) (Gibco, Life Technologies™, Carlsbad, California, USA) supplemented with 10% FBS and 5% penicillin/streptomycin (Gibco, Life Technologies™, Carlsbad, California, USA), and incubated at 37°C with 5% CO_2_.

### Downregulation of CD98hc and production of lentivirus

Plasmid construction for targeting human CD98hc was performed as described by Poettler et al. 14. One µl of the plasmid of interest was transfected into competent XL-Gold cells according to the heat-shock method. Single colonies were inoculated in LB medium with ampicillin and incubated overnight. Plasmid purification was performed using the Maxi Plasmid Preparation Kit (Qiagen) according to the manufacturer's protocol. HEK-293T cells were transfected using the calcium-phosphate method as described earlier (14). Supernatant from HEK-293T cells was collected after 48 and 72 hours, and filtered (0,45 µm). Virus was stored at -80°C. Pancreatic cancer cell lines were infected with 200 µL of the supernatant in the presence of polybrene 10 µg/mL (Sigma-Aldrich, St. Louis, Missouri, USA). Puromycin (Gibco, Life Technologies™, Carlsbad, California, USA) was used to select infected cells after 72 hours. The medium was changed after 24 hours. In order to generate stable CD98hc/PANC-1 and CD98hc/BxPC-3 (scrambled sh(small hairpin)RNA), cells were grown in the presence of puromycin 5 µg/mL (Gibco, Life Technologies™, Carlsbad, California, USA). Downregulation of CD98hc (shCD98hc) was confirmed by flow cytometry (data not shown).

#### Proliferation assay

PANC-1 and BxPC-3 cells were seeded at 120,000 cells per well of 6-well culture plates and incubated at 37°C. After 24, 48 and 72 hours, cells were harvested with trypsin/EDTA and counted using a hemocytometer after the addition of trypan blue (Gibco, Life Technologies™, Carlsbad, California, USA) to identify and exclude dead cells.

### Analysis of the capacity for self-renewal and anchorage-independent growth

#### Sphere formation assay

PANC-1 and BxPC-3 cells were detached from the cell flasks using accutase (Gibco, Life Technologies™, Carlsbad, California, USA) and single cell suspension was controlled under the microscope. 1,000 of PANC-1 or BxPC-3 cells per well of a low-attachment 6-well plate were resuspended in DMEM-F12 (ham's nutrient mixture F12) supplemented with epidermal growth factor (PeproTech, Cranbury, New York, USA) (120 ng/mL), insulin (5 µg/mL), transferrin (2.75 μg/mL) and puromycin (5 µg/mL). BxPC-3 cells were additionally supplemented with ROCK (rho-kinase) inhibitor (Cayman Chemical, Ann Arbor, Michigan, USA). After 14 days, formed spheres were counted under the microscope. Primary and secondary spheres were dissociated by adding accutase (Gibco, Life Technologies™, Carlsbad, California, USA). Cells were resuspended every 5 minutes and the single-cell suspension was examined under the microscope.

#### Soft colony agar assay

Bottom agar medium was prepared using 2x DMEM (Gibco, Life Technologies™, Carlsbad, California, USA), FBS and 1.8% soft agar to reach a final concentration of 0.3% agar. Agar-coated 6-well plates were incubated for 30 min at room temperature. 1,250 cells of each cell line per well were resuspended in a top agar solution. The agar concentration of the final solution was 0.6%. After 2 weeks, colonies were counted under the microscope.

### Statistical analysis

Statistical analyses were performed using IBM SPSS Statistics 25.0 (SPSS Inc., Chicago, IL, USA). All *in vitro* experiments were repeated three times. Kolmogorov-Smirnov test was used to assess the normal distribution of the data. Normally distributed data are presented as mean ± SD, whereas data that were not normally distributed are given as median [min-max]. Differences between normally distributed parameters of two independent groups were compared with the independent Student's t-test. Survival data were available for 74 patients. For statistical analysis of the relationship between CD98hc immunostaining and tumor grade, stage and overall survival, the cohort was divided into four groups: (1) patients with CD98hc expression in the tumor as well as in the adjacent tissue, (2) patients with no CD98hc expression (3) patients with CD98hc expression in the tumor alone and (4) patients with CD98hc expression only in the adjacent tissue. The effect of CD98hc on overall survival in patients with pancreatic ductal adenocarcinoma was established by a Kaplan-Meier plot and further analyzed using a Cox proportional hazards regression model. A *p*-value *<*.05 was considered statistically significant.

## Results

### CD98hc expression in human fetal and adult healthy pancreatic tissue and pancreatic ductal adenocarcinoma

**Figure 1:**
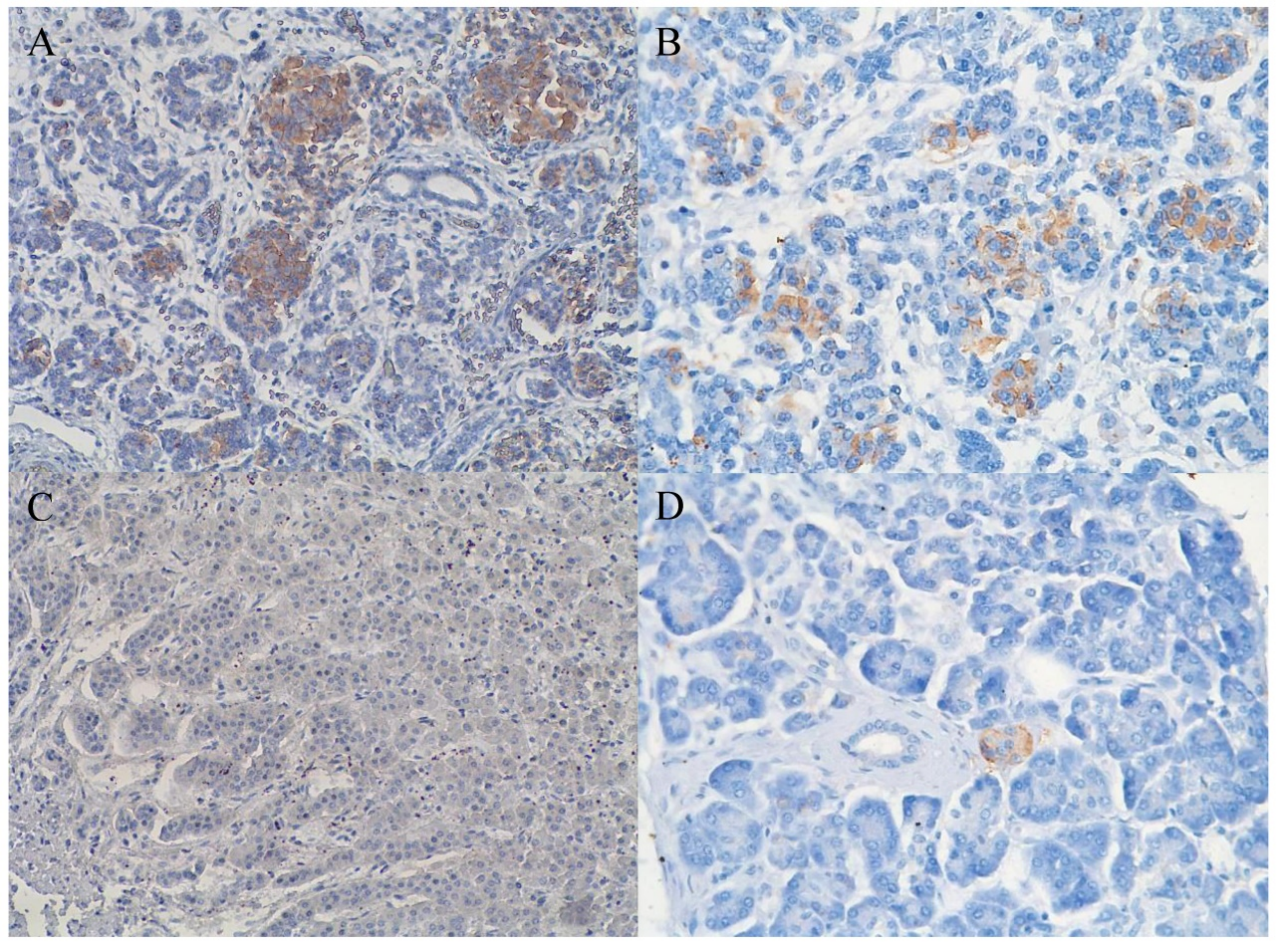
Expression of CD98hc in acinar and centroacinar cells (A) and in islet cells (B) of healthy fetal (20 weeks) pancreatic tissue as identified by the brownish areas. Lack of expression of CD98hc in acinar cells of adult human pancreatic tissue (C); expression of CD98hc in islet cells of healthy adult pancreatic tissue (D); original magnification 40x.

**Figure 2:**
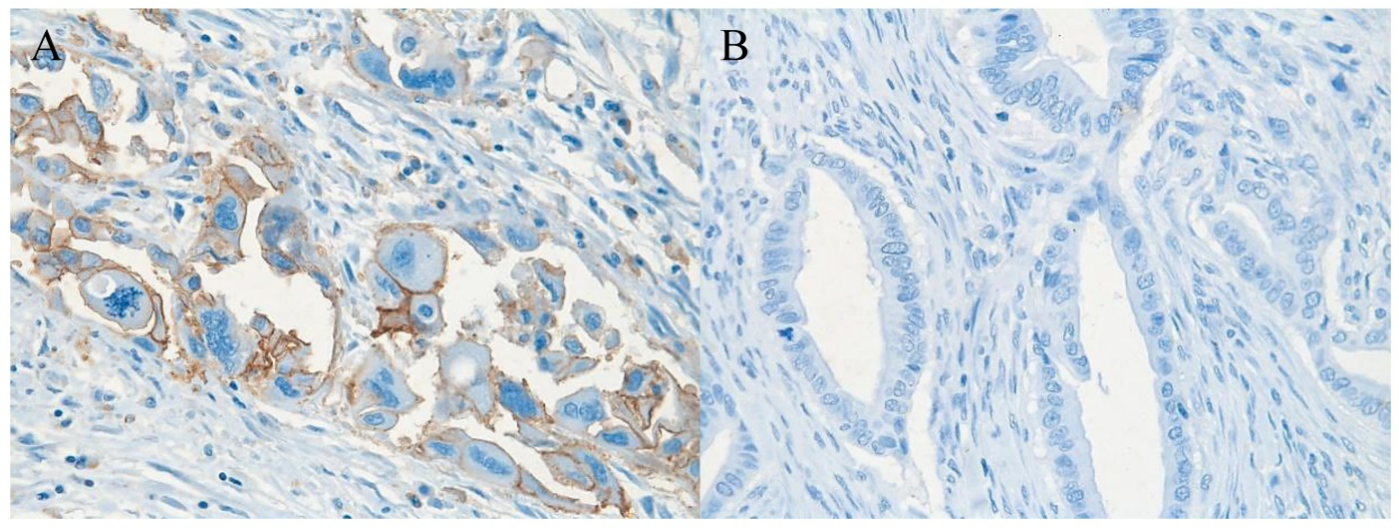
Pancreatic ductal adenocarcinoma tissue with (A) (brownish color) and without (B) expression of CD98hc; original magnification 40x.

**Figure 3:**
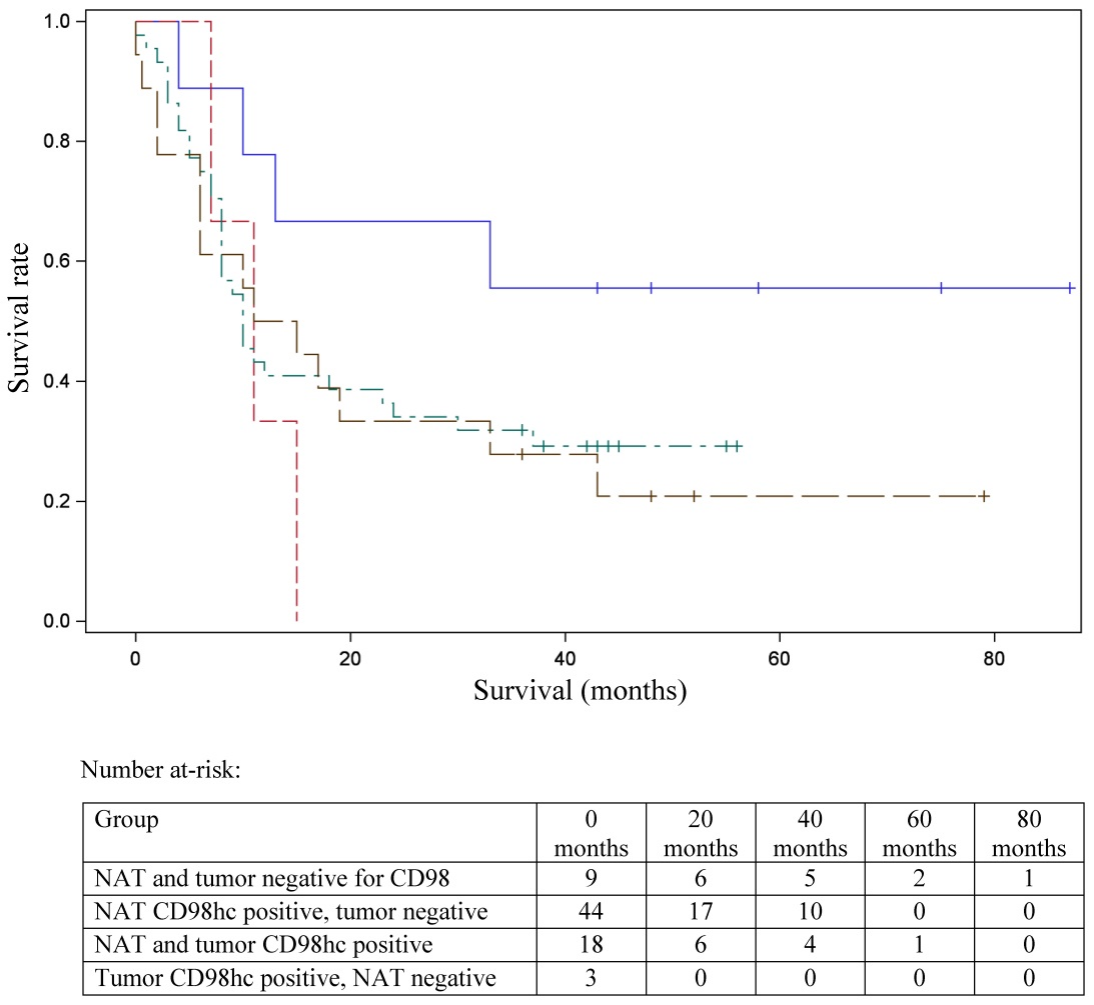
Kaplan-Meier curve for overall survival stratified according to the patterns of CD98hc expression. Survival data were available for 74 patients. NAT and tumor negative (blue line); NAT CD98hc positive, tumor negative (green); NAT and tumor CD98hc positive (brown line); tumor CD98hc positive, NAT negative (red line). NAT: normal adjacent tissue.

In 40% of human fetal pancreatic tissue, expression of CD98hc was found in acinar and centroacinar cells (Figure 1A), and in islet cells (Figure 1B). In adult pancreatic tissue, immunohistochemical positivity for CD98hc was detected in only 1% of samples in acinar cells (Figure 1C) and in islet cells (Figure 1D). Thirteen percent of investigated pancreatic ductal adenocarcinomas expressed CD98hc in acinar cells and 20% expressed it in the islet cells embedded in tumor tissue (Figure 2). Expression of CD98hc in tumor tissue was not associated with a particular tumor stage or grade. Furthermore, its expression in pancreatic ductal adenocarcinoma was not a significant prognostic factor for overall survival (hazard ratio (HR) 0.64 (p=0.271) for CD98hc expression in normal adjacent tissue (NAT) and HR=0.77 (p=0.375) for CD98hc expression in tumor tissue) as illustrated by the Kaplan-Meier curves of four groups categorized according to CD98hc expression (Figure 3).

### Effects of CD98hc downregulation on cell proliferation, self-renewal and anchorage-independent cell growth

**Figure 4:**
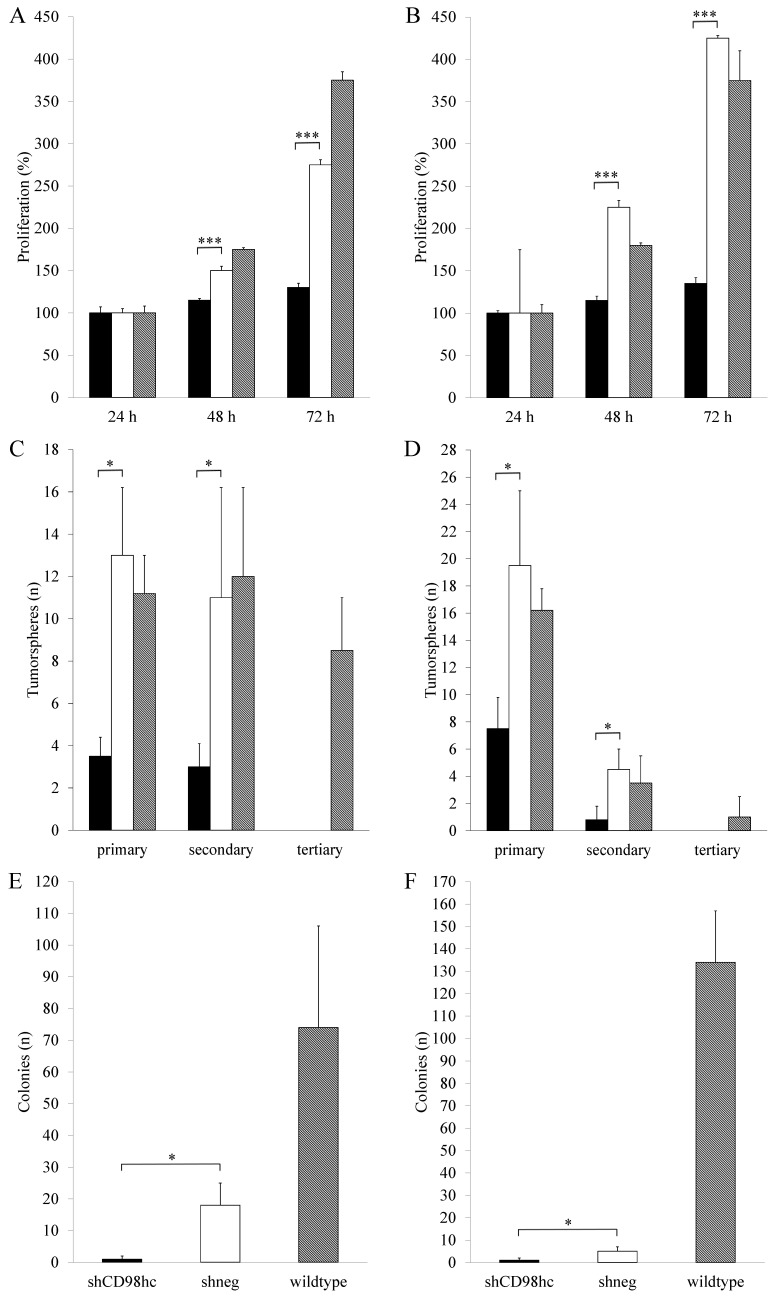
Downregulation of CD98hc inhibits proliferation in PANC-1 (A) and BxPC-3 (B) cancer cells, impaired tumorsphere formation in PANC-1 (D) and BxPC-3 (D) cancer cells and impaired colony formation in soft agar in PANC-1 (E) and BxPC-3 (F) cancer cells. Black bars: shCD98hc cells, white bars: shneg cells, grey bars: wildtype cells. Comparison between shCD98hc cells and shneg cells was analyzed using the Student's t-test. Three independent experiments were performed in triplicates. *P < 0.05 and ***P < 0.001.

Downregulation of CD98hc expression via shRNA in the pancreatic cancer cell lines PANC-1 and BxPC-3 resulted in a significant (p<0.05, in both cases, after 48h and 72h) inhibition of cell proliferation in shCD98hc cells as compared to control cells (transduced with shRNA, shneg) and wildtype cells in both cell lines (Figure 4A and 4B).

In shCD98hc cells of both cell lines (PANC-1 and BxPC-3), the capacity for self-renewal, as reflected by tumorsphere formation, was significantly lower than in control (shneg) cells (p<0.05 in both cell lines) in terms of primary sphere formation. In both cell lines, this capacity further decreased with the first passage to form secondary spheres (Figure 4C and 4D) and neither shCD98hc cells nor shneg cells could form tertiary tumorspheres.

Assessment of anchorage-independent cell growth by employing a soft agar colony forming assay revealed that shCD98hc of PANC-1 and BxPC-3 cells had a significantly lower colony-forming ability than shneg cells (p<0.05) (Figure 4E and 4F).

## Discussion

This study was conducted to evaluate the expression of CD98hc in pancreatic ductal adenocarcinoma tissue and to elucidate its functional role in cancer cell behavior to provide data for a potential new therapeutic approach in this malignancy. In accordance with the pivotal role of CD98hc in normal cell proliferation and development, our data revealed expression of CD98hc in 40% of human fetal pancreatic acinar and centroacinar cells, but in only 1% of the investigated pancreatic tissue of healthy adults. This suggests that CD98hc expression in pancreatic tissue is gradually diminished with maturity, as also found in fibroblasts 29. While CD98hc expression does not seem to be essential for mature healthy cells, the lack of CD98hc in mouse embryonic stem cells blocks cell proliferation *in vivo* 6 and CD98hc deficiency in mice produces an embryonic lethal phenotype 33. Furthermore, CD98hc knockout fibroblasts fail to survive standard culture conditions due to iron-dependent oxidative (non-apoptotic) cell death (ferroptosis), attributable to the loss of CD98hc-xCT, which provides cyst(e)ine for intracellular synthesis of glutathione, thereby sustaining cellular redox homeostasis 23,24,34. As a consequence, CD98hc-deficient cells present with accumulation of reactive oxygen species and intracellular amino acid imbalance with a dramatic increase in cationic amino acids and neutral amino acids but reduced levels of branched-chain and aromatic amino acids, which results in nutritional and replicative stress, and impaired cell proliferation and cell growth 23,24,34.

In contrast to healthy adult cells, expression of CD98hc is found in a wide variety of different cancer cells such as renal cell cancer 35, non-small-cell lung cancer 36, pulmonary neuroendocrine tumors 37, squamous cell carcinoma of the lung, thymic epithelial tumor 38, oropharyngeal squamous cell carcinoma 39, prostate cancer 40, gastric carcinoma 41 and cancer of the tongue 42. Our data show that up to 20% of pancreatic ductal adenocarcinomas express CD98hc in acinar cells (13%) and islet cells (20%) embedded in tumor tissue. Although the expression of CD98hc in tumor tissue of treatment-naive patients was not associated with a particular tumor stage or grade, there was a trend towards longer overall survival of pancreatic cancer patients without CD98hc expression as compared to those with immunohistochemical positivity. This may be due to the pivotal function of CD98hc as a transmembrane transporter for a broad spectrum of substrates including all essential amino acids 23. As such CD98hc plays a key role in sustaining amino acid and nucleotide availability, glucose cellular nutrition and redox homeostasis for cell cycle progression and cell growth 21, i.e. promoting cancer cell proliferation an growth. Data of patients with pancreatic cancer support this suggestion because the expression of CD98hc-LAT1 on resected cancer tissue correlates with a poor prognosis 43. Moreover, CD98hc is involved in the activation of TRPV4 channels 30, which mediate cyclic strain-induced endothelial cell reorientation 44 and restore normal angiogenesis in tumors by modulating the Rho/Rho kinase pathway 45. Due to this crucial role of CD98hc in cell proliferation, signaling and metabolism as well as formation of metastases, it may serve as a specific molecular target for the development of new anticancer therapeutic strategies in the future.

Why CD98hc expression in acinar and centroacinar cells is downregulated with maturity, and upregulated in some cases of pancreatic cancer, remains speculative. Expression of CD98hc may be one of the first steps towards malignant cell transformation in the pancreas, leading to altered metabolism, higher cell proliferation, altered extracellular matrix and inflammation. On the other hand, cancer cells probably alter their microenvironment in such a way that CD98hc expression is induced in the adjacent tissue. Indeed, an epithelial cancer model has shown that CD98hc significantly modulates tumor microenvironment and cellular responses 4, but data on similar effects in pancreatic cancer are currently not available.

Our finding of CD98hc expression in islet cells may be linked to the well-accepted, but not yet fully elucidated association between diabetes and pancreatic cancer. Data from the Italian Pancreatic Cancer Study Group reported that 23% of 720 cancer patients had an accompanying diagnosis of diabetes 46. Further, a meta-analysis of 11 case-controlled studies, which included data from 2,546 pancreatic cancer patients, showed an approximately 2-fold increase in the risk of pancreatic cancer in long-standing diabetes (>5 years) 47. However, it is difficult to establish causality from data that demonstrate an association of diabetes and pancreatic cancer 48.

In addition to our finding of CD98hc expression in pancreatic cancer tissue, specific *in vitro* experiments were carried out to better understand the contribution of CD98hc to pancreatic cancer cell behavior and tumor malignancy. These experiments found that downregulation of CD98hc expression significantly inhibited proliferation, self-renewal and anchorage-independent growth in the pancreatic cancer cell lines PANC-1 and BxPC-3, suggesting an important role of CD98hc in malignant transformation in pancreatic cancer. Among the drivers of malignant cell growth, integrin-interacting CD98hc has been found to act as an oncogene-stimulating molecule leading to anchorage-independent growth within CHO cells 49. Further evidence of the crucial role of CD98hc in malignant cell growth was provided by an embryonic stem cell model lacking CD98hc: While wildtype cells formed teratomas, CD98hc-deficient cells showed reduced proliferation, cell survival and tumor growth 6. Major steps of malignant cell behavior, such as tumor cell spreading, transmigration, proliferation, cell survival and formation of metastases, were found to be dependent on the cytoplasmic integrin-interacting domain of CD98hc; whereas the amino acid transport activity was primarily associated with cancer cell proliferation 14.

Although this study has several limitations such as the sample size for the immunohistochemistry analysis and the lack of *in vivo* studies, our data provide evidence that CD98hc is upregulated in human pancreatic ductal adenocarcinoma and suggest an important role of CD98hc in malignant transformation in this disease. Even though our findings show a trend towards longer overall survival of pancreatic cancer patients without CD98hc expression, the hypothesis of CD98hc expression being a prognostic factor in this diesase is limited by unavailability of treatment-related data after surgery (e.g. chemotherapy, which might have had a significant influence on overall survival. Further studies are needed to get better insights into the role of CD98hc in the pathogenesis and malignant cell transformation in pancreatic cancer, which may be the basis for the development of new targeted therapeutic approaches for this disease in the future.

## Conclusion

A remarkable percentage of pancreatic ductal adenocarcinomas express the transmembrane protein CD98hc, which is crucial for cell proliferation and development due to its function as a coreceptor of ß-integrins, amplifying intracellular integrin-dependent downstream signaling, and as a transporter of branched-chain and aromatic amino acids. Downregulation of CD98hc *in vitro* significantly inhibits proliferation, self-renewal and anchorage-independent growth in the pancreatic cancer cell lines PANC-1 and BxPC-3, suggesting an important role of CD98hc in cell behavior and malignant cell transformation. Thus, CD98hc may serve as a molecular target for potential new therapeutic approaches in pancreatic cancer in the future.

## Figures and Tables

**Table 1:** Baseline characteristics of the investigated cases of pancreatic ductal adenocarcinoma

	Cases n (%)	Female n (%)	Male n (%)	Age in years median [min-max]
Total	222 (100)	144 (55)	78 (45)	62 [34-85]
Normal adjacent tissue (NAT)	141 (64)	85 (60)	78 (40)	61 [36-85]
NAT and tumor negative for CD98hc	9	7 (78)	2 (22)	59 [50-75]
NAT CD98hc positive, tumor negative	44	29 (66)	15 (34)	62 [34-81]
NAT and tumor CD98hc positive	18	7 (39)	11 (61)	65 [43-85]
Tumor CD98hc positive, NAT negative	3	1 (33)	2 (67)	56 [36-59]

Total				
Grade	G1	G2	G3	Unknown
n (%)	27 (12)	179 (81)	15 (6.5)	1 (0.5)
Stage	T1	T2	T3	Unknown
n (%)	9 (4)	166 (75)	44 (20)	3 (1)
	N0	N1	N2	-
n (%)	127 (57)	75 (34)	20 (9)	-
	M0	M1	-	-
n (%)	214 (96)	8 (4)	-	-

## Abbreviations

ATCC: American Type Culture Collection
CD98: cluster of differentiation 98 heavy chain
DMEM: Dulbecco's Modified Eagle's Medium
DNA: deoxyribonucleic acid
EDTA: ethylenediaminetetraacetic acid
FBS: fetal bovine serum
HAT: heteromeric amino acid transporter
HR: hazard ratio
LAT: L-type amino acid transporter
mTORC1: mammalian target of rapamycin complex 1
NAT: normal adjacent tissue
PBS: phosphate buffer saline
RNA: ribonucleic acid
shCD98hc: small hairpin cluster of differentiation 98 heavy chain
shRNA: small hairpin RNA
ROCK: rho-kinase
TRPV4: transient receptor potential vanilloid 4
xCT: cystine/glutamate transporter
